# Establishment and application of cricothyrotomy in vivo

**DOI:** 10.1186/s12909-023-04558-w

**Published:** 2023-08-07

**Authors:** Fengxiang Song, Cailing Han, Bin Liu, Yuxue Qiu, Haitao Hou, Xiaoqiong Yan, Liqin Deng

**Affiliations:** Department of Anesthesiology and Perioperative Medicine, General Hospital of Ningxia Medical University, Yinchuan, Ningxia 750004 China

**Keywords:** Airway, Anesthesiology, Animal model, Cricothyrotomy, Training

## Abstract

**Background:**

Cricothyrotomy is a procedure performed to establish an airway in critical airway events. It is performed only rarely and anesthesiologists are often unprepared when called upon to perform it. This study aimed to simulate cricothyrotomy using pig larynx and trachea models to help anesthesiologists master cricothyrotomy and improve the ability to establish cricothyrotomy quickly.

**Methods:**

The porcine larynx and trachea were dissected and covered with pigskin to simulate the structure of the anterior neck of a human patient. An animal model of cricothyrotomy was established. Forty anesthesiologists were randomly divided into four groups. Each physician performed three rounds of cricothyrotomy, and recorded the time to accomplish each successful operation. After training the cricothyrotomy procedure, a questionnaire survey was conducted for the participating residents using a Likert scale. The participants were asked to score the utility of the training course on a scale of 1 ((minimum) to 5 ((maximum).

**Results:**

Through repeated practice, compared with the time spent in the first round of the operation (67 ± 29 s), the time spent in the second round of the operation (47 ± 21 s) and the time spent in the third round of the operation (36 ± 11 s) were significantly shortened (*P* < 0.05). Results of the survey after training were quite satisfied, reflecting increased the ability of proficiency in locating the cricothyroid membrane and performing a surgical cricothyrotomy.

**Conclusion:**

The porcine larynx and trachea model is an excellent animal model for simulating and practicing cricothyrotomy, helping anesthesiologists to master cricothyrotomy and to perform it proficiently when required.

## Background

Even with adequate airway assessment before administering general anesthesia, the emergency event of “Can't Intubate Can't Oxygenate” (CICO) cannot be avoided entirely after induction of general anesthesia. If ventilation cannot be guaranteed at this time, it will lead to rapid hypoxic brain damage and even patients’ death. Cricothyrotomy is the preferred invasive intervention in response to CICO emergencies with unanticipated difficult airways. The 2022 American Society of Anesthesiologists (ASA) Practice Guidelines for the Management of Difficult Airways emphasizes that if an invasive airway (such as cricothyrotomy) is required, it should be established as soon as possible and ensured by a trained physician with sufficient experience in invasive airway techniques [1]. Therefore, in addition to mastering the practices and procedures of difficult airway management, anesthesiologists are advised to strengthen training at ordinary times so they can respond promptly and effectively when difficult airways are encountered. However, CICO adverse events in clinical anesthesia are uncommon, and it is not possible to master this technology in routine clinical practice. Mastering this first-aid technology proficiently and resolving the airway crisis remains a troublesome issue when training qualified anesthesiologists. This study intends to establish an animal model of cricothyrotomy, aiming to train anesthesiologists to master cricothyrotomy and to improve anesthesiologists' ability to deal with CICO through repeated practice, helping to ensure the safety of patients' lives.

## Materials and methods

### Cricothyrotomy animal model preparation

**Fig. 1:**
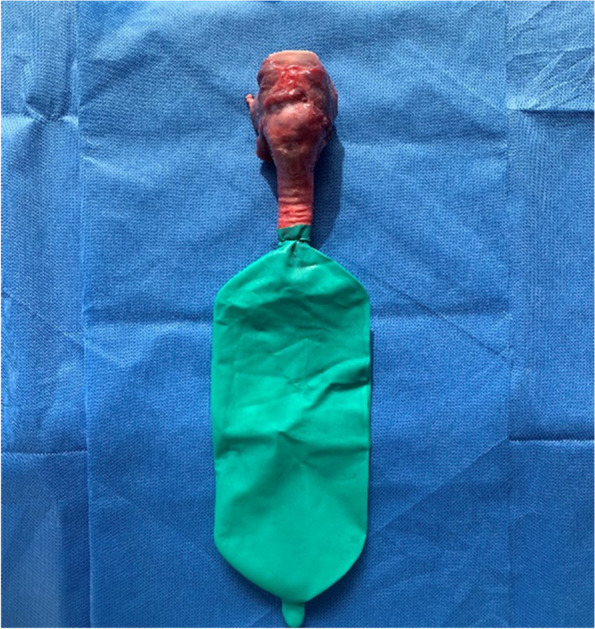
Pig larynx and trachea specimens after complete separation

**Fig. 2:**
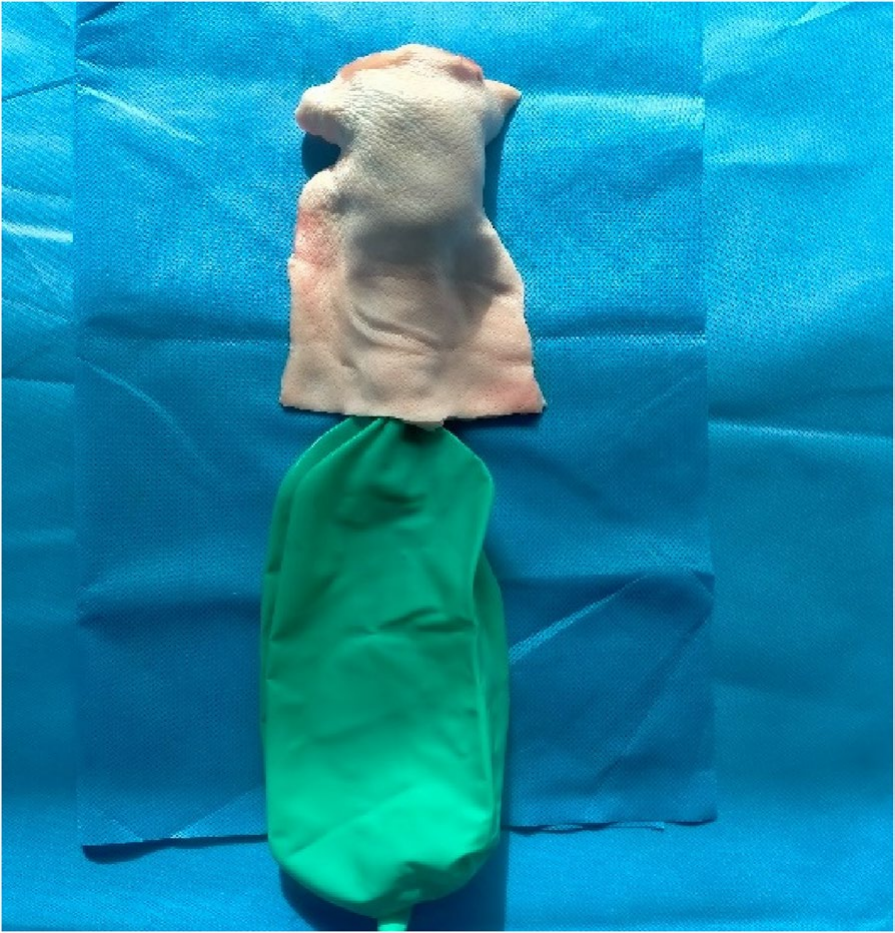
Laryngeal and trachea specimens covered with pigskin

A prominent local slaughterhouse was selected, and the slaughtered mature pigs were placed in a supine position. The limbs were fixed, and the mandibular soft tissue and thorax were cut open in sequence to expose the epiglottis cartilage, thyroid cartilage, cricoid cartilage, and trachea. The epiglottis cartilage, thyroid cartilage, cricoid cartilage, and trachea were isolated, and a 20 cm*30 cm piece of pig skin was peeled off (hair removed, no subcutaneous tissue). Cover the airway specimen with pig skin in preparation for cricothyrotomy. A total of 120 samples were prepared (Fig. 1, Fig. 2).

### Cricothyrotomy training

#### Training subjects

Forty residents in the Anesthesiology Department of Ningxia Medical University.

#### Training location

Clinical Skills Training Center, General Hospital of Ningxia Medical University.

#### Instrument preparation

Disposable sterile gloves, No. 10 surgical blade, bougie with a curved head, and 6.0 mm endotracheal tube with cuff.

#### Theoretical training

Media explanation of the 2022 version of ASA difficult airway management practice guidelines, knowledge updates, and the critical points of cricothyrotomy.

#### Operational training

**Fig. 3:**
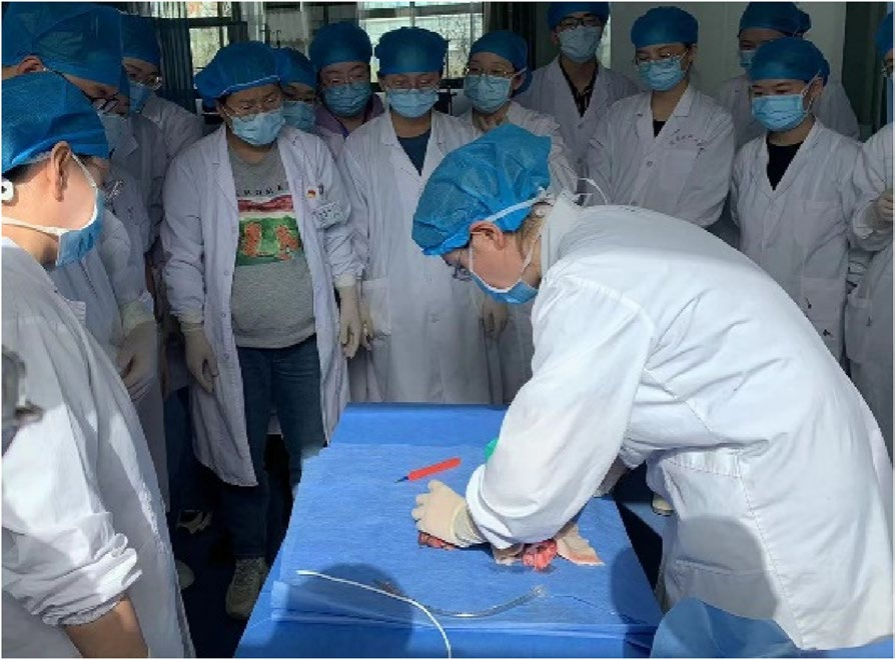
Experienced senior anesthesiologists demonstrate standard implementation of cricothyrotomy to resident physicians in the Department of Anesthesiology

An experienced senior anesthesiologist will demonstrate how to standardize the implementation of cricothyrotomy. (Fig. 3).

### Implementation of cricothyrotomy

**Fig. 4:**
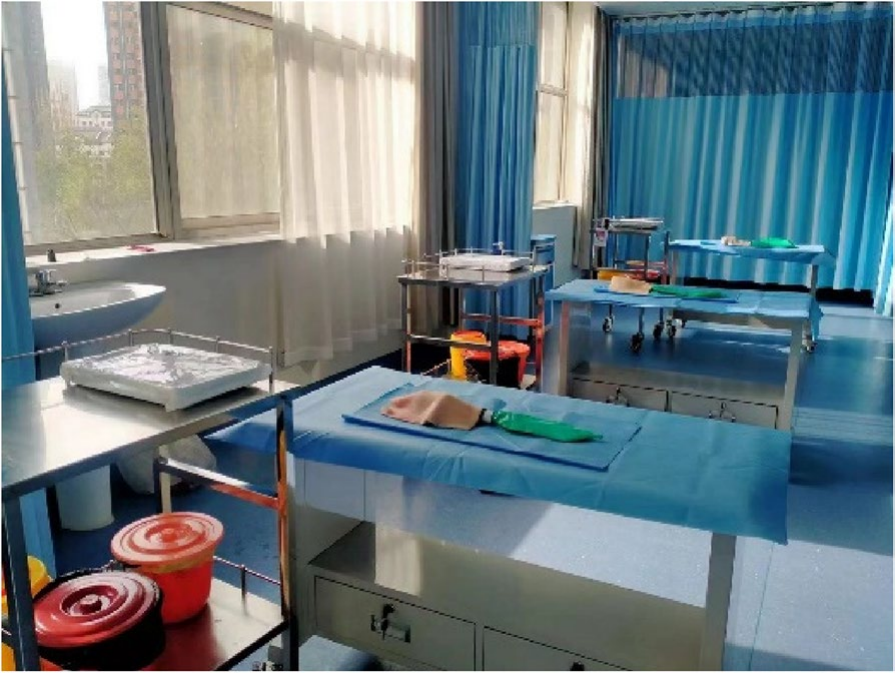
Training site

**Fig. 5:**
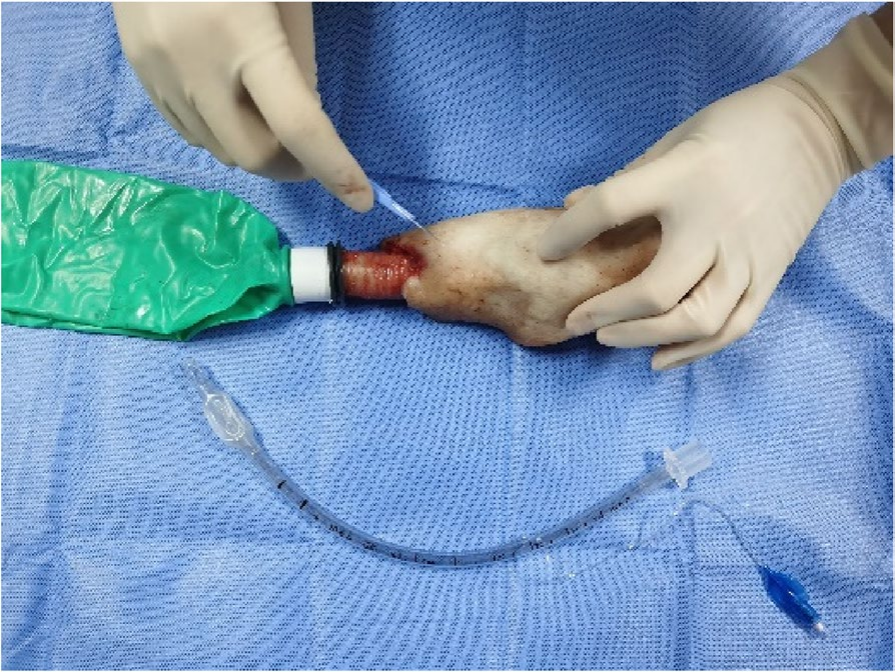
Experienced senior anesthesiologists use their fingers to distinguish between laryngeal cartilage and Cricothyroid membrane

**Fig. 6:**
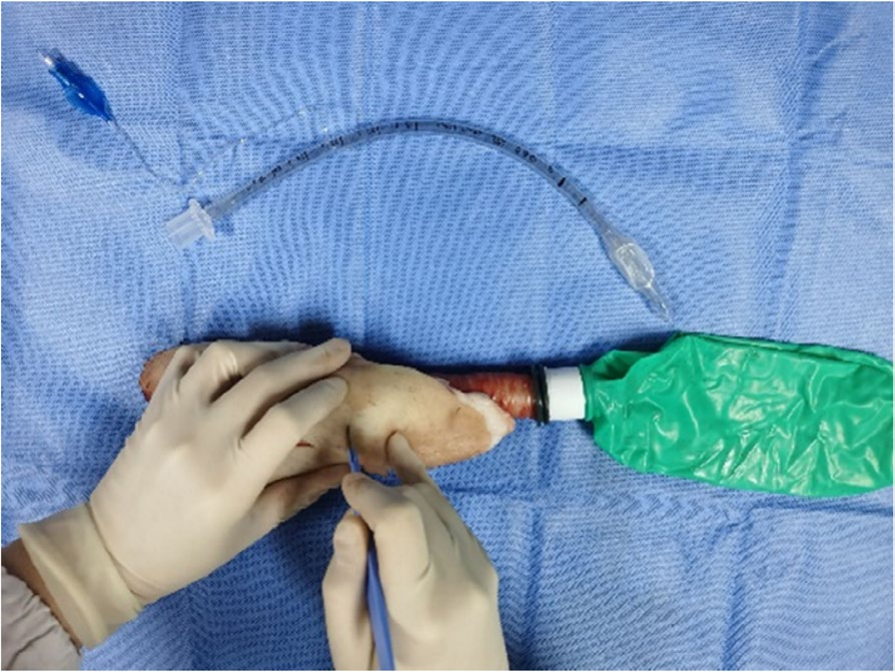
Lateral incision for open cricothyrotomy technique

**Fig. 7:**
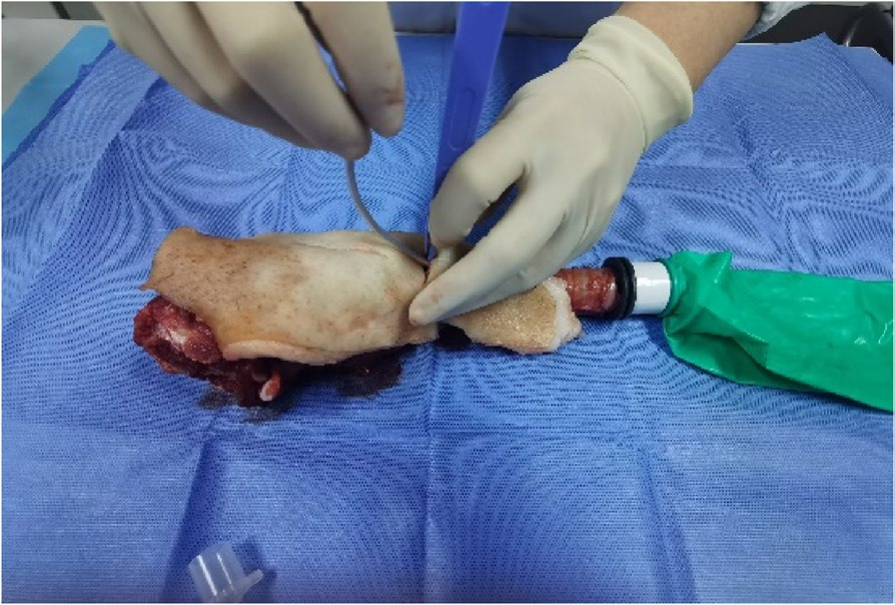
Bougie insertion

**Fig. 8:**
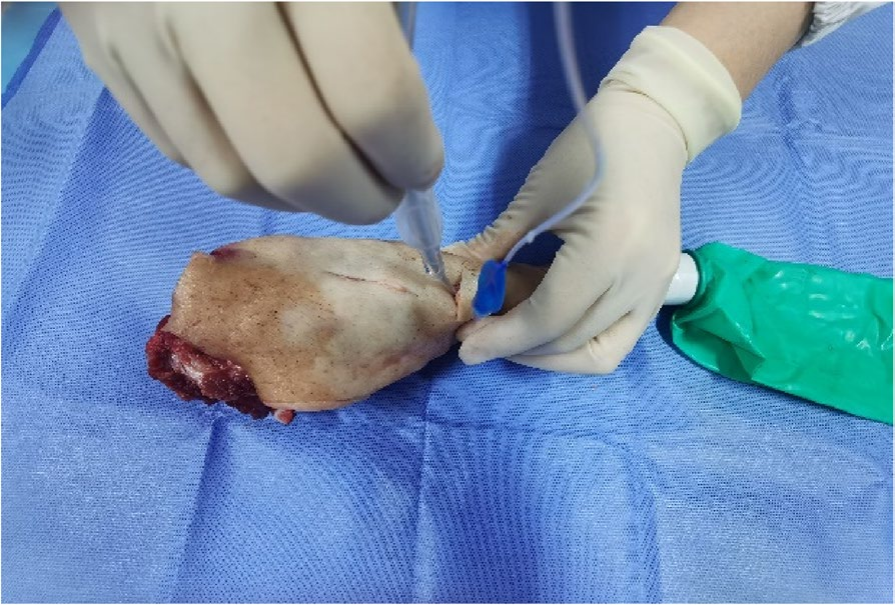
Size 6.0 mm endotracheal tube is advanced over bougie, which has been placed through open cricothyrotomy

Each physician completed three rounds of operations according to the standard and recorded the successful operation time and first-time success rate of cricothyrotomy. First, the porcine cricothyrotomy model was placed on the operating table face up (Fig. 4). Then the operator, wearing gloves, stood on the right side of the model and fixed (stabilized) the trachea in front of the neck using the thumb and middle finger of the left hand. Then the index finger of the left hand was slid up and down to determine the position of the cricothyroid membrane (Fig. 5). The operator held the No. 10 scalpel blade firmly in the right hand, with the blade facing the operator, and incised the cricothyroid membrane vertically, downward, and horizontally (Fig. 6). The scalpel 90° was rotated clockwise to enlarge the cutting-edge. Keeping the scalpel still, it was replaced with the left hand, holding it steadily while inserting the bougie into the trachea 10–15 cm along with the right hand (Fig. 7). The scalpel was then removed, and a 6.0 mm endotracheal tube was advanced into the trachea by a bougie. Finally, the endotracheal tube cuff was inflated, the pressure was less than 30 cm H_2_O, and a simple respirator was connected to simulate assisted ventilation (Fig. 8). The observation of reciprocal movement in the respiratory balloon attached to the distal end of the tracheal tube, in synchrony with the utilization of a manual resuscitator for simulated assisted ventilation, served as a reliable indicator of successful endotracheal intubation.

### Evaluation of the cricothyrotomy model

After completing the cricothyrotomy procedure, a questionnaire survey was conducted among the participating residents using a Likert scale. The participants were asked to rate the utility of the training course on a scale of 1 ((minimum) to 5 ((maximum).

### Statistical analysis

SPSS 26.0 software (IBM SPSS, Chicago, IL, USA) was used for all statistical analysis. Measurement data that conformed to normal distribution were expressed as mean ± standard deviation ($\overline{x }$ ± SD). Comparisons within the group was performed by repeated measures data analysis of variance (ANOVA), and comparisons between groups were evaluated using one-way ANOVA. P < 0.05 was established as statistical significance.

## Results

### Comparison of timed and success rated for three rounds of cricothyrotomy by physicians

Compared with the operation time of the first round, the operation time of each physicians’ second and third rounds was significantly shorter (*P* < 0.05). Compared with the operation time of the second round, the operation time of the third round of each group was significantly shorter (*P* < 0.05) (Table 1). In the first round of the operation, one person in the first group and one in the fourth group failed on the first time, and the first-time success rate of the other groups was 100%.

**Table 1:** Comparison of operation times in three rounds of each physician in four groups $\left(s, \overline{x } \pm s\right)$

Number of Procedures	Participants	Time (Seconds)
First round	40	67.45 ± 29.05^bc^
Second round	40	46.65 ± 20.91^a^
Third round	40	36.51 ± 11.36^b^
F-value		10.585
*P*-value		< 0.001

^a^*P* < 0.05: Compared with the first round of operation time

^b^*p* < 0.05: Compared with the second round of operation time

^c^*p* < 0.05: Compared with the second round of operation time

### Survey on the cricothyrotomy model among participants

The results of survey after training were highly positive, indicating strong endorsement from the participating residents and an increased proficiency in performing the cricothyrotomy procedure.(Table2).

**Table 2:** Survey on the cricothyrotomy model among anesthesiologists

Question	Mean (SD) Evaluation Score
This exercise provided valuable practical experience that cannot be obtained through plastic models	4.85(0.5)
This exercise offered an opportunity to simulate the procedure in a realistic scenario, allowing me to become familiar with the steps and process of cricothyrotomy	4.76(0.69)
This exercise enhanced my understanding of the theoretical knowledge and practical application of cricothyrotomy	4.98 (0.16)
This exercise strengthened my ability to perform cricothyrotomy rapidly and accurately in emergency situations	4.5 (0.9)
This exercise improved my proficiency and skills in using relevant equipment and tools	4.85 (0.5)
This exercise fostered my interest in further research and learning about cricothyrotomy	4.98 (0.16)

^a^A five-point Likert scale was used, with a value of 5 denoting “strongly agree” and a value of 1 denoting “strongly disagree”

## Discussion

Results of the present study show that the porcine larynx and trachea model reasonably simulates a human model of cricothyroidotomy, providing a suitable model for training anesthesiologists. Among 40 trainees performed the cricothyrotomy procedure three consecutive times, the success rate of the first round of cricothyrotomy was 95%, and the success rate of the second and third rounds of cricothyrotomy was 100%. The results of survey after training indicated the strong endorsement from the participating residents in performing the cricothyrotomy procedure by using the porcine larynx and trachea model.

The incidence of CICO emergency airway has been significantly reduced in recent years due to the rapid development of medical technology and medical equipment, especially the wide application of various airway management tools such as video laryngoscope, laryngeal mask, and flexible electronic endoscopic [2]. In routine clinical practice, emergencies requiring cricothyrotomy are infrequent, and most anesthesiologists are relatively inexperienced in performing the procedure [3]. The Fourth National Survey in the United Kingdom reported that the failure rate of cricothyrotomy performed by anesthesiologists was as high as 64% [4]. Since anesthesiologists are unfamiliar with the cricothyrotomy procedure and lack clinical experience, they will inevitably lack confidence in establishing a surgical airway in emergency airway management, so they are likely to hesitate due to fear of failure, causing them to miss the best rescue opportunity [5, 6]. Therefore, it becomes essential to choose a suitable cricothyrotomy practice model that will help improve the success rate of anesthesiologists in performing cricothyrotomy in emergencies, and effectively improve the success rate of emergency airway rescue [7].

Results of the present study showed that repeated practice of using the porcine larynx and trachea model significantly reduced the time for resident physicians in the Department of Anesthesiology to perform cricothyrotomy, suggesting that anesthesiologists can quickly master cricothyrotomy after training. Similarly, Shetty et al. reported that the time to complete a cricothyrotomy was significantly shorter after each participant's consecutive cricothyrotomy attempts, which is consistent with our findings [8]. The 2022 ASA practice guidelines for difficult airway management emphasize that emergency cricothyrotomy is the last treatment option for CICO. Although it is rarely used as an option for resolving critical airway events, anesthesiologists should still practice diligently to ensure that emergency cricothyrotomy can be performed at any time [1]. Therefore, it is crucial to train anesthesiologists to master cricothyrotomy using animal models, to calmly deal with critical emergency situations and difficult airway events, helping to ensure the safety of patients' lives with adequate preparation via clinical practice teaching methods.

Plastic models are often used in clinical teaching, but they do not effectively simulate human tissues. Under natural conditions, the failure rate of cricothyrotomy is high, making it essential to establish a solid model for simulation teaching of cricothyrotomy. Due to the similarity of the porcine anatomy to that of human beings, porcine models are widely used in medical research and teaching [9–13]. The present study constructed pig larynx and trachea models by which to practice cricothyrotomy with the ultimate goal of improving clinicians' ability to deal with emergency airways.

Compared to previous studies using porcine trachea models [11–13], this study employed fresh porcine skin to cover the model, aiming to better simulate the sensation of human skin. This improvement allowed trainees to have a more realistic experience during simulated procedures, enhancing the practicality and realism of the training. Unlike artificial materials or other animal skins, the trainees said that the use of porcine skin in this study provided a closer resemblance in appearance and texture to human skin, enabling trainees to better perceive the tactile sensations and haptic feedback during actual operations. Another innovation of this model is its disposable nature. In contrast to the reusable models used in previous research [11], this study utilized disposable models to avoid the traces and damages associated with repeated use, thereby maintaining consistency and accuracy in each training session. This design ensured that every trainee could undergo training under the same conditions, improving the comparability and reliability of the training outcomes.

However, there are several limitations in this study. Firstly, the distance from the skin to the trachea in our porcine trachea models remains relatively consistent, which fails to simulate the varying subcutaneous tissue thickness observed in patients of different body weights. Secondly, our trainees consisted solely of first-year and second-year residents who lacked prior clinical experience with cricothyrotomy, making it challenging to ascertain whether this model effectively replicates human cricothyrotomy. Additionally, our survey questionnaire did not gather feedback from each student. In conclusion, the porcine larynx and trachea model is an excellent animal model for simulating and practicing cricothyrotomy, which will help anesthesiologists master cricothyrotomy and perform it proficiently in routine clinical practice.

## Data Availability

The datasets used and analyzed during the current study are available from the corresponding author on reasonable request.
